# Immunogenicity and efficacy of oral vaccines in developing countries: lessons from a live cholera vaccine

**DOI:** 10.1186/1741-7007-8-129

**Published:** 2010-10-04

**Authors:** Myron M Levine

**Affiliations:** 1 Center for Vaccine Development, University of Maryland School of Medicine, Baltimore, MD 21201, USA

## Abstract

Oral vaccines, whether living or non-living, viral or bacterial, elicit diminished immune responses or have lower efficacy in developing countries than in developed countries. Here I describe studies with a live oral cholera vaccine that include older children no longer deriving immune support from breast milk or maternal antibodies and that identify some of the factors accounting for the lower immunogenicity, as well as suggesting counter-measures that may enhance the effectiveness of oral immunization in developing countries. The fundamental breakthrough is likely to require reversing effects of the 'environmental enteropathy' that is often present in children living in fecally contaminated, impoverished environments.

## 

Vaccines represent the epitome of a preventive strategy to control disease [1,2]. In the individual, they confer direct protection and, if high enough immunization coverage of a population is achieved, unimmunized people may also be protected, indirectly, through 'herd immunity' [3,4]. The strategic use of some vaccines, such as measles and polio vaccines, has interrupted indigenous transmission of those diseases in entire regions of the globe [5-8]. And one disease, smallpox, has been completely eradicated from the human population through the epidemiologically sound use of smallpox vaccine [9,10].

In developing countries, where two-thirds of the world's population live, infectious diseases cause most of the mortality among children under 5 years of age [11] and constitute major health problems in older children and adults. Vaccines are among the most promising interventions to diminish the burden of specific infections in populations in developing countries [12-15].

## The special advantages of oral vaccines

Oral vaccines are particularly attractive for immunizing populations in developing countries for several reasons. First, contaminated needles and syringes are major problems both for health workers and for environmental safety in many developing countries where there is a high prevalence of HIV and hepatitis B and C [16-18]. Because they obviate the need for needles and syringes, oral vaccines allow less qualified health workers to carry out immunization. Second, the simple logistics of oral vaccines are highly compatible with mass immunization campaigns [19,20]. Lastly, in most societies both adults and children generally prefer an oral vaccine to a parenteral injection.

Despite the attractions of oral vaccines for developing countries, many oral vaccines, both live and non-living, have proven to be less immunogenic or less protective when administered to infants, children or adults living in low socioeconomic conditions in less-developed countries than they are when used in industrialized countries (Table 1). Thus, there is a poorly understood 'intestinal barrier' to successful immunization of people in less developed countries who receive oral vaccines. Here, I review this phenomenon, provide examples and possible explanations, and offer suggestions for establishing the basis of the phenomenon.

**Table 1:** Oral vaccines associated with diminished immunogenicity or efficacy in developing country populations

Oral vaccine	Target ages at which diminished immunogenicity or protection was observed	Geographic locations where observed	References
Sabin polio vaccine strains	Infants, toddlers, preschool children, school-age children	India, sub-Saharan Africa	[25-33]
RIT 4237 rotavirus	Infants	Gambia	[36]
Rotashield rotavirus vaccine (10^4 ^plaque forming unit dosage)	Infants	Brazil and Peru	[37,125]
Rotarix attenuated rotavirus	Infants	Malawi, South Africa, Bangladesh	[38]
Rotateq pentavalent attenuated rotavirus	Infants	Ghana, Kenya, Mali	[39]
MMU18006 (monovalent Rhesus rotavirus strain)	Infants	Pakistan	[40]
CVD 103-HgR live cholera strain	24-59 months; 5-9 years; adults	Indonesia, Thailand, Peru, Ecuador	[41-43,64]
Dukoral non-living cholera vaccine (killed *V. cholerae* O1 plus B subunit)	1-12 years	Nicaragua	[45]
SC602 attenuated *Shigella flexneri *strain	Toddlers and school age children	Bangladesh	[46,47]

## Immune responses to oral vaccines in developing countries

### A prototype: Sabin oral polio vaccine

The prototype oral vaccine is the Sabin attenuated strains trivalent polio vaccine (tOPV), which eliminated transmission of wild-type polioviruses in the Americas [21], the Western Pacific [22] and Europe [22] and has been the linchpin of the Global Polio Eradication Initiative [23,24]. Of the three poliovirus serotypes (types 1 to 3), tOPV has interrupted transmission of type 2 poliovirus globally since 1999.

Despite the remarkable milestones of disease control achieved with tOPV, it has been recognized since the 1960s that tOPV seems to be poorly and inconsistently immunogenic in some developing country populations [25-31]. Diminished immunogenicity has been a particularly vexing problem in the states of Uttar Pradesh and Bihar in India [32,33], from which wild-type polioviruses have been disseminated to other states in India and elsewhere in South Asia. By late 2005, the average child under 5 years of age in Bihar and Uttar Pradesh had received about 15 doses of OPV compared with about 10 doses for children of the same age elsewhere in India [34]. However, because of diminished immunogenicity, only an estimated 71% of children under age 5 years in these two states were successfully immunized against polio, compared with 85% of children elsewhere in India [34]. Diminished immune responses in children in these areas of India are correlated with poor sanitation, a high prevalence of diarrheal illness at the time of vaccination, competing enteric viruses and competition of type 2 with types 1 and 3 vaccine viruses [34]. Type 2 Sabin OPV strain colonizes the intestine better and is considerably more immunogenic than types 1 or 3. For this reason tOPV is formulated to contain less type 2 virus than the other two serotypes (ratio of 10^6^:10^5^:10^5.8 ^infectious units per dose) [35] to try and mute its dominance. Nevertheless, in some developing country populations, including Uttar Pradesh and Bihar, it was necessary to change from use of tOPV in mass campaigns to the selective use of monovalent type 1 and 3 vaccines or to bivalent type 1+3 vaccine to improve immune responses to these serotypes and to interrupt transmission [34,35].

### Other oral vaccines with diminished immunogenicity or efficacy

Table 1 lists various oral vaccines for which data from clinical trials have demonstrated either a diminished immune response or lower efficacy in developing countries than in industrialized country populations. Besides Sabin polio vaccine [25-31], these oral vaccines include rotavirus vaccines [36-40], CVD 103-HgR live cholera vaccine 4144, B subunit-inactivated *Vibrio cholerae *whole cell combination vaccine [45] and SC602 live *Shigella flexneri *2a vaccine [46,47]. Thus, the oral vaccines implicated include both viral and bacterial and both live and non-living vaccines. Moreover, the phenomenon has been observed in all age groups, from young infants to adults. To maximize the protective effects that can be achieved with oral vaccines in developing countries, it will be important to understand why immune responses and efficacy tend to be lower in such target populations than in populations in industrialized countries.

### Oral vaccines in young infants

Some common factors probably contribute to lowering the immunogenicity and efficacy of live oral vaccines among people of all ages in developing countries. However, there are special confounding factors in the case of young infants. In that age group, there is likely to be some level of immunity due to maternal serum IgG antibodies transferred *in utero*, and to breast milk, which contains maternal secretory immunoglobulin A (sIgA) antibodies [48], immune cells and non-specific protective factors, such as lactoferrin [49,50] and oligosaccharides [51]. These both provide protection against pathogens and modulate responses to vaccines.

Early studies with tOPV, RIT 4237 rotavirus vaccine [36], tetravalent rhesus reassortant rotavirus vaccine at the 10^4 ^plaque forming unit dosage level [52] and other candidate rotavirus vaccine strains [40] indicated a barrier to oral immunization. Two new rotavirus vaccines, Rotarix, the monovalent human G1P[8] strain attenuated by multiple passages in tissue culture [53], and Rotateq, a pentavalent vaccine based on reassortant bovine rotavirus expressing human rotavirus surface proteins G1 to 4 and P[8,54], have been shown to be safe, immunogenic and highly protective against severe rotavirus gastroenteritis in large-scale, placebo-controlled efficacy trials in infants in North America, Europe and South America. However, when tested in efficacy trials in Africa and Asia, these two vaccines showed much lower efficacy [38,39]. The level of efficacy tended to correlate with the level of development of the population in which the vaccines were tested. Although this may reflect environmental influences such as competing enteric viral, bacterial or protozoan infections, it is likely that higher titers of breast milk IgA and maternally derived serum IgG antibodies against rotavirus also played a role in the places where vaccine efficacy was lowest.

### Oral vaccines in older age groups

Various oral vaccines have demonstrated diminished immunogenicity or efficacy in older age groups, including in pre-school and school-age children and adults. By focusing on vaccines in these age groups, it is possible to identify and study environmental and host factors without the confounding effects of breast milk and maternal antibodies. The vaccine that has been most intensively studied for these factors is live oral cholera vaccine strain CVD 103-HgR, a genetically engineered vaccine derived from a wild-type *V. cholerae *O1 classical biotype, Inaba serotype strain. In this vaccine, 94% of the gene encoding the enzymatically active A subunit of cholera toxin has been deleted and a gene encoding mercury ion resistance inserted into the hemolysin A locus as an indelible marker [55-57]. Our experience, and that of other groups, with CVD 103-HgR is reviewed below to illustrate how factors associated with diminished immunogenicity to oral vaccines in developing country populations can be identified and examined in order to devise ways to overcome the barrier.

## The CVD 103-HgR live oral cholera vaccine as a paradigm

Two O serogroups of *V. cholerae*, O1 and O139, can cause epidemics of cholera gravis. *V. cholerae *O1 is by far the more important as O139 infections are found in just a few areas of Asia (where they are responsible for only a few percent of cases) and O139 has not been reported from Africa. Two biotypes of *V. cholerae *O1 exist, El Tor and classical, although presently only El Tor strains are prevalent. Recently, highly virulent El Tor strains have emerged that produce classical biotype cholera enterotoxin. Within each biotype of O1 are found two main serotypes, Inaba and Ogawa. For a cholera vaccine to be a useful public health tool, it must protect against both serotypes and biotypes. In North American adults, a single oral dose of about 5 × 10^8 ^colony forming units (cfu) of CVD 103-HgR elicits significant (four-fold or greater) rises in serum vibriocidal antibody (that is, seroconversion) in over 90% of those vaccinated [56,58] and vaccine organisms are excreted by about 25% [56,58]. A single dose of CVD 103-HgR significantly protects North Americans against cholera caused by *V. cholerae *O1 of either classical or El Tor biotype and either Inaba or Ogawa serotype [56,59-61].

**Figure 1:**
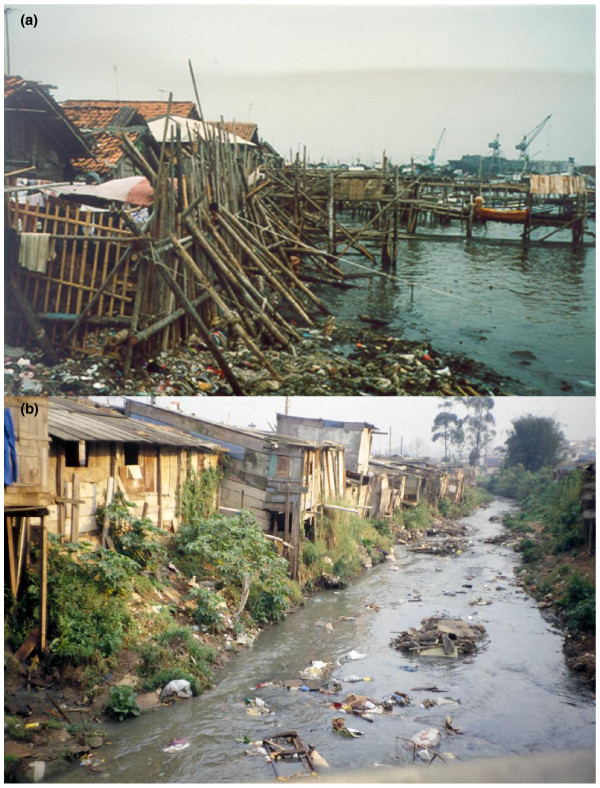
**Cholera-endemic living conditions**. **(a) **Conditions of ramshackle housing, poor sanitation and widespread fecal contamination prevalent in North Jakarta in the early 1990s when phase 2 pediatric clinical trials with CVD 103-HgR live oral cholera vaccine were carried out. **(b) **Similar conditions of inadequate housing, lack of sanitation and fecally contaminated surface waters in a favella (periurban slum) in São Paulo, Brazil of the type in which environmental enteropathy was first described by Fagundes Neto. Photograph kindly provided by Ulysses Fagundes Neto, Universidade Federal de São Paulo.

The first study in a developing country examining the safety and immunogenicity of CVD 103-HgR was carried out among young adult students on a Research Isolation Ward at Mahidol University, Bangkok, Thailand [62], with immunogenicity results closely resembling those seen in healthy North Americans and Europeans. Therefore, bolstered by the promising results of this (small) trial, a pediatric study was initiated in children 5 to 9 years of age living in a squalid, cholera-endemic slum in North Jakarta, Indonesia [41] (Figure 1a). In these children, the 5 × 10^8 ^cfu dose of CVD 103-HgR that had been so highly immunogenic in North American [56,58] and Swiss [63] adults and higher socioeconomic level Thai university students [62] elicited significant increases in serum vibriocidal antibody in only 16% of the 5- to 9-year-old Indonesian children living in poverty [41]. This was the first demonstration that diminished immunogenicity in developing country situations could also be encountered with oral bacterial vaccines [41], as had been recognized for many years with oral viral vaccines.

Fortunately, by administering a tenfold higher dose of CVD 103-HgR (5 × 10^9 ^cfu) to children 5 to 9 years of age in North Jakarta, it was possible to achieve a high rate of seroconversion [41]. Moreover, the few non-responders were shown to be children who had high baseline titers of serum vibriocidal antibody and therefore were apparently already immune to cholera. Results with children 2 to 4 years of age living in the same area were similar [64]. In studies with both adults and children, those who did not seroconvert had a significantly higher baseline vibriocidal titer than those who did seroconvert [41-43,64], indicating that such individuals are already immune and their serum titers are not typically boosted by vaccination.

It is worth noting that oral vaccines are expected to elicit locally produced intestinal antibodies and tests of serum antibodies do not detect these local antibodies. It is thus conceivable that intestinal antibodies may have increased in these studies. Indeed, it has long been surmised that rises in serum vibriocidal antibodies serve as a proxy for the elicitation of immune responses in the small intestine. It is also possible that some individuals may have baseline intestinal immunity not reflected by an elevated serum vibriocidal titer

A series of additional immunogenicity studies with CVD 103-HgR were undertaken in Asia [42], South America [43,65-69] (after the El Tor pandemic of cholera reached that continent in 1991) and Africa [70]. From this composite of clinical trials, we identified several factors that modulated the magnitude of the vibriocidal antibody response. The first of these is prior exposure to *V. cholerae *O1, resulting in high baseline vibriocidal antibody titers: titers are not usually boosted in individuals with high baseline titers [41-43,64]. The second factor is blood group O: people with blood group O (a well recognized host risk factor for development of cholera gravis [71,72]) mount stronger serum vibriocidal responses [66], especially if immunologically naïve - that is, with no prior exposure to *V. cholerae *O1. The third factor is socioeconomic level: populations in under privileged conditions show lower antibody titers, independent of blood group or prior contact with *V. cholerae *O1 [42,43]. The fourth factor is small bowel bacterial overgrowth (SBBO) [44] that often accompanies environmental enteropathy (see below) [73,74], which in turn is related to living in poverty-associated fecally contaminated conditions. The fifth factor is heavy infection with intestinal helminths [75,76]. The sixth factor is HIV status: although the rates of seroconversion are not significantly different, the antibody titers of HIV-positive individuals are significantly lower than those of HIV-negative individuals [70].

To achieve high seroconversion rates of vibriocidal antibody in Peruvian and Thai adults living in under privileged conditions, as with Indonesian children living in poverty, it was necessary to give a tenfold higher dose (5 × 10^9 ^cfu) of CVD 103-HgR [41-43] than the dosage level (5 × 10^8 ^cfu) that was consistently immunogenic in North Americans and Europeans [56,58]. This 10^9 ^cfu dosage level was also well tolerated and immunogenic in pre-school children [64,67], toddlers [68] and infants as young as 3 months of age [68].

## The role of environmental enteropathy and small bowel bacterial overgrowth

**Figure 2:**
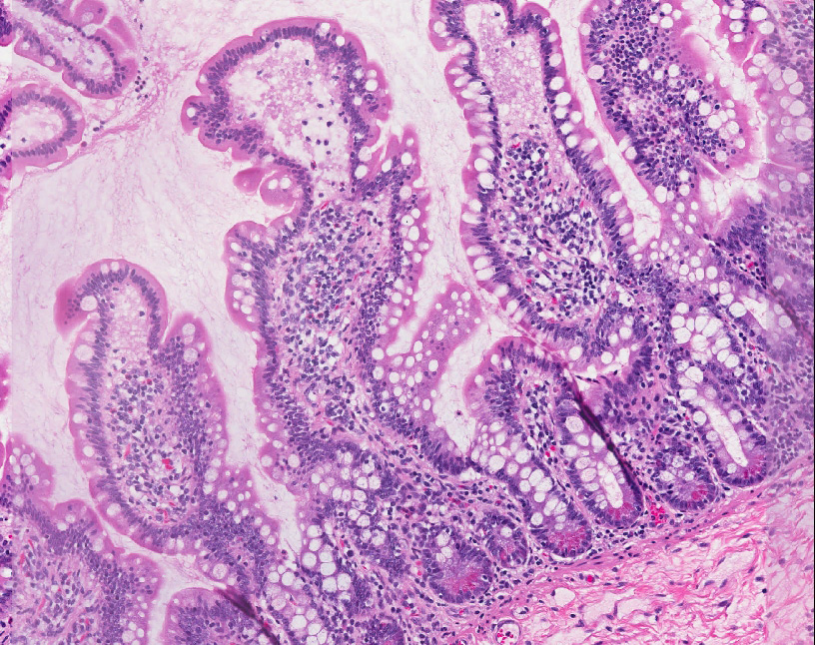
**Normal intestinal mucosa**. Biopsy of the second portion of the duodenum of an 8-year-old US child showing normal histology of the intestinal mucosa. Long, finger-like villi and relatively shallow crypts are evident. The villi are populated with columnar epithelial cells (enterocytes) that have brush borders containing enzymes for digestion and absorption; mucus-producing goblet cells are interspersed among the enterocytes. Less than 20 intraepithelial lymphocytes per 100 enterocytes are present. Photomicrograph kindly provided by Steven Czinn, University of Maryland Medical Center.

The proximal small intestines of healthy children and adults who live in relatively pristine environments in industrialized countries show only modest bacterial loads, whether measured by aerobic and anaerobic culture or by molecular techniques based on analysis of 16S rDNA sequencing of DNA from appropriate clinical specimens [77,78]. Common known bacterial genera identified include *Streptococcus*, *Veillonella*, *Neisseria*, *Gemella*, *Rothia *and *Hemophilus*; in contrast, fecal genera, such as are found in the colon or terminal ileum (where microbiota densities are enormous), are uncommon. Duodenal biopsies show that the mucosa of healthy children is characterized histologically by the presence of long, finger-like villi, ample columnar epithelial cells, a crypt to villus ratio of 1:3 or 1:4, less than 25 intra- epithelial lymphocytes per 100 columnar cells and only a moderate number of mononuclear cells in the lamina propria (Figure 2). In contrast, the gut of children living in poverty in developing countries often reflects their continual exposure to fecally contaminated environments, and many such children have SBBO and 'environmental enteropathy' [73,74,79-81].

**Figure 3:**
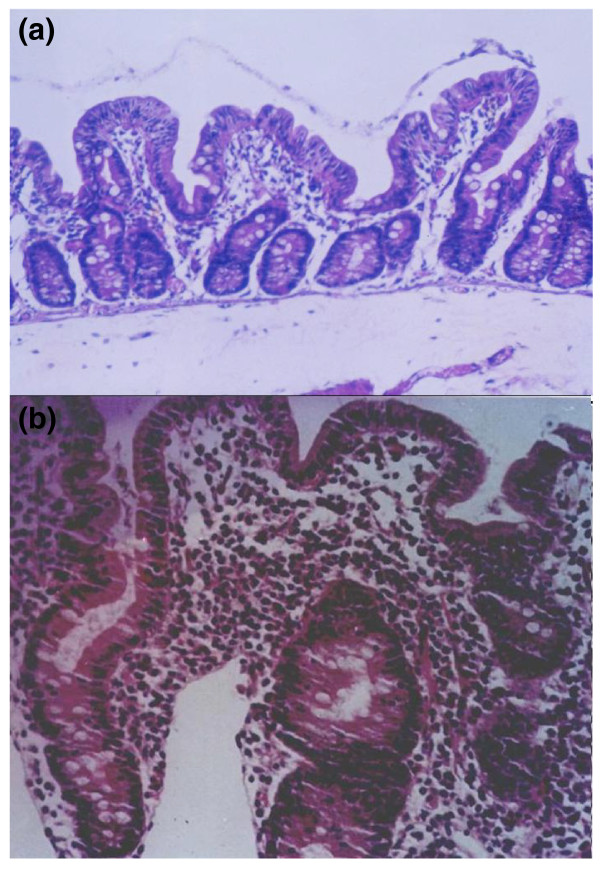
**Intestinal mucosa showing environmental enteropathy**. **(a) **Biopsy of the distal duodenum of a 36-month old Brazilian child with environmental enteropathy. Moderate villous atrophy is seen with blunted, flat villi and enterocytes that seem somewhat cuboidal rather than columnar. Elongated crypts can be seen, which result in an abnormal crypt to villus ratio. Most prominent is the striking increase in the number of lymphocytes and plasmacytes present in the lamina propria. **(b) **Biopsy of the distal duodenum of another Brazilian pre-school age child with environmental enteropathy. The changes are as described for (a) but the villous blunting and hypercellularity within the lamina propria are even more prominent. Photomicrographs kindly provided by Ulysses Fagundes Neto, Universidade Federal de São Paulo.

The term environmental enteropathy was coined by Fagundes Neto [73,74,80] to describe a syndrome that includes non-specific histopathological and functional changes of the small intestine in children of poor families living in conditions lacking basic sanitary facilities and chronically exposed to fecal contamination (Figure 1b). The prominent histopathological features of environmental enteropathy include blunted villi, abnormal crypt to villus ratio, an increased number of intraepithelial lymphocytes and a marked increase of lymphocytes and plasmacytes in the lamina propria (Figure 3a, b). A key feature of environmental enteropathy is the presence of SBBO that includes fecal bacterial species usually restricted to the terminal ileum and colon. Another salient feature of environmental enteropathy is its disappearance over time following the individual's transfer to a clean environment characterized by improved food hygiene and modern sanitation [80]. Environmental enteropathy is similar (and perhaps identical) to the syndrome of 'tropical enteropathy' described by Linden- baum *et al. *[82] in US Peace Corps volunteers who lived among indigenous populations for about two or more years, often in conditions characterized by heavy fecal contamination. The intestinal lesions observed in most of these young adults also slowly returned to normal several months after the volunteers returned to the USA [82].

The presence of SBBO can be detected by having fasting children ingest the disaccharide lactulose and detecting H_2 _in expired breath by gas chromatography at various time points; measurements are typically made at baseline and 15, 30, 40, 60, 90 and 120 minutes after ingestion of the lactulose [44,80,83]. Human intestinal enzymes cannot cleave lactulose but bacterial enzymes can. Therefore, the detection of H_2 _in 'small bowel' specimens - that is, those taken 15 or 30 minutes after lactulose ingestion -indicates SBBO [44,80,83]. The advantage of the lactulose breath H_2 _test is that it is amenable to field studies involving hundreds of children [44,80,83].

When the relationship between SBBO and vibriocidal response to CVD 103-HgR was investigated in 202 fasting Chilean schoolchildren aged 5 to 9 years who had lactulose breath H_2 _tests one day before ingesting CVD 103-HgR [44], florid small bowel overgrowth was observed in 10 out of 178 analyzable children (5.6%), and logistic regression analysis showed that increased peak breath H_2 _at 'small bowel' time points was associated with diminished vibriocidal antibody seroconversion, as was the interaction of peak breath H_2 _and weight (*P *= 0.02) [44].

SBBO could blunt the immune response to CVD 103-HgR by the production of short chain fatty acids (such as butyric and propionic acids) [84] or other small molecules [85] that directly inhibit the *V. cholerae *O1 vaccine, thereby markedly decreasing the actual dose of vaccine organisms. Immune responses to cross-reacting surface antigens of intestinal flora may also blunt the vibriocidal response to CVD 103-HgR. Alternatively, the effect may be indirect. Individuals with SBBO typically have abnormal intestinal architecture [73,74] and increased lymphocytes and plasmacytes in the mucosa [73,74], perhaps with activated T cells [86]. One possible explanation is that under conditions of repetitive exposure to fecal contamination, the innate immune system of the child's gut is maximally activated and in a pro-inflammatory state. This may constitute an important non-specific defense in the proximal small intestine, rendering it generally hostile to incoming bacterial (and viral and protozoal) pathogens. Indeed, this may be what allows many children to survive repeated exposures to enteric pathogens. By extrapolation, attenuated bacterial or viral oral vaccines must also face this hostile, innate immune system-activated ecologic niche when they reach the proximal small intestine, resulting in inhibition of the vaccine organisms and poor induction of specific adaptive immune responses. Live vaccines might then, instead of activating the innate immune system to enhance adaptive immune responses (as would happen in an industrialized country gut), be destroyed by an already highly activated innate immune response.

Supporting this hypothesis is the observation that although people in developing countries show moderate or high rates of seroconversion following ingestion of the 10^9 ^cfu dosage level of CVD 103-HgR, they also show significantly lower rates of excretion of the vaccine strain [41,43,64,67,68]. It is increasingly recognized that normal gut homeostasis (including gut immunity) involves 'crosstalk' among the microbiota present in the outer layer of the mucus biofilm covering the mucosa, enterocytes and cells of the immune system [87-91].

An alternative explanation for the decreased immune responses is that the mononuclear cell hypercellularity observed in the mucosa of patients with environmental enteropathy may indicate altered regulatory T cell and dendritic cell function that contributes to dampening of immune responses [81,92,93]. It is possible that environmental enteropathy modifies the proximal small bowel ecology so much that it begins to resemble the colon not only in its microbiota but in the immunological functioning of its mucosa (immune structures commonly form in chronically infected mucosa). Collectively, these ideas may be considered the obverse of the 'hygiene hypothesis' - the widely popularized notion that the increasing prevalence of allergies in industrialized countries reflects a failure to develop the normal regulatory balance of the adaptive immune response when exposure to environmental pathogens is limited [94-96].

Further evidence of a role for intestinal infection in the diminished vibriocidal antibody response to the CVD 103-HgR vaccine has been gathered in studies on school age children in rural Ecuador with documented heavy helminthic (*Ascaris lumbricoides*) infection [75,76]. These children were randomly allocated to receive two courses of an anti-helminthic (albendazole) or placebo and were then immunized with a single 5 × 10^8 ^cfu dose of CVD 103-HgR [75]. For children of blood group O, there was no difference in the vibriocidal responses observed in the albendazole versus the placebo groups. However, for children of non-O blood groups, those treated with albendazole had a significantly higher vibriocidal antibody response than those given placebo.

## Extrapolating to other oral vaccines

Some of the factors that seem to contribute to the diminished immunogenicity of CVD 103-HgR may be relevant to other oral live vaccines. Live oral *Shigella flexneri *2a candidate SC602 was reactogenic in North American volunteers when ≥ 10^6 ^cfu were ingested [97]. However, ingestion of a lower, better tolerated dose (10^4 ^cfu) was followed by heavy excretion, strong immune responses and protection against experimental challenge with wild- type *S. flexneri *2a [97]. In contrast, when tested in a phase 1 trial in Bangladeshi toddlers, neither vaccine excretion nor immune responses were observed following ingestion of 10^4^, 10^5 ^or 10^6 ^cfu of SC602 [47].

Licensed live oral typhoid vaccine Ty21a may be a notable exception. This live oral vaccine does not elicit strong (that is, high titer) serum antibody responses [98,99] but does stimulate intestinal IgA antibodies [100] as well as robust B [101] and T cell-mediated [102-105] immune responses, and appropriate formulations and immunization schedules of Ty21a have conferred significant protection on school-age children for up to 7 years in large-scale, randomized, controlled efficacy trials in Egypt [106], Chile [107-110] and Indonesia [111]. This may be a function of the way *Salmonella *Typhi interacts with the small intestinal mucosa, as this organism effectively targets the M cells that overlie gut-associated lymphoid tissue [112,113] and is then readily taken up by the underlying dendritic cells and macro- phages. Thus, Ty21a easily and rapidly gains access to inductive sites of the immune system. This may hold true for several markedly more immunogenic modern recombinant *S*. Typhi single-dose vaccine candidates that are in development [114-117].

## A way forward

Now that the poor response of many people in developing countries to a variety of oral vaccines has been documented and widely recognized, a consensus is emerging that action should be initiated to study the phenomenon, with a view to counteracting the factors responsible for the intestinal barrier [118]. With respect to the role of SBBO and the alterations of the intestinal mucosa that characterize environmental enteropathy, there is much to be done. A first step should be to separate any direct inhibitory role of the bacterial flora itself, whether in mucus-associated biofilm or in the lumen, from the broader defects that may be consequent on the altered architecture and function of the intestinal mucosa. A controlled trial should be undertaken to determine whether temporarily eliminating SBBO with oral antibiotics before oral immunization enhances immune responses to the vaccine [119]. If this has a positive effect, then non-antibiotic interventions (such as competing probiotic bacteria) should be studied.

*Giardia *infections are highly prevalent among children in developing countries but are increasingly recognized not to be associated with either diarrhea or adverse nutritional consequences [120]. Nevertheless, *Giardia *may have an impact on mucosal integrity and function that may diminish responses to oral vaccines. Thus, I would advocate a randomized, placebo-controlled trial in which half the participants receive metronidazole to eradicate *Giardia *before oral vaccination.

If eliminating SBBO with antibiotics or *Giardia *with metronidazole has no impact, then ways should be explored to repair the integrity and function of the intestinal mucosa. Vitamin A modestly improved immune responses to type 1 poliovirus vaccine but did not enhance serum vibriocidal responses to a killed oral cholera vaccine [121]. Two studies investigated zinc supplementation and responses to a non-living oral cholera vaccine; one [121] showed slight improvement of vibriocidal responses following zinc supplementation, whereas the other [122] reported suppression. This suggests that further evaluation of zinc is needed. Alanyl-glutamine may improve gut integrity in patients with environmental enteropathy [123]. Lastly, modifying the innate immune system of the gut in relation to oral vaccination should be studied with increased stimulation (vaccine plus a mucosal adjuvant such as *Escherichia coli *double mutant LT [124]) to counteract possible tolerance, or with dampening of the innate immunity (vaccine plus a suppressive agent) to determine which approach, if any, has an ameliorating effect.
